# Immunological evaluation of an mRNA vaccine booster in individuals fully immunized with an inactivated SARS‐CoV‐2 vaccine

**DOI:** 10.1002/ctm2.875

**Published:** 2022-06-09

**Authors:** Xingli Xu, Yun Liao, Guorun Jiang, Weiguo Yao, Suqin Duan, Kang Xiao, Xuefeng Ding, Heng Zhao, Yujian Zhang, Aihua Zhang, Jingsi Yang, Yanchun Che, Jun Zhang, Fanfan Zhao, Xiaopin Ma, Zhimei Zhao, Pingfang Cui, Xiaolei Yang, Xiaorui Lin, Wei Cai, Jiao Yan, Zhenqing Yang, Heng Qiu, Jing Zhang, Lei Huang, Mingyun Shen, Guofeng Zhao, Li Yu, Dandan Li, Shengtao Fan, Ying Zhang, Lichun Wang, Licun He, Fei Dong, Wenbo Xu, Hangwen Li, Qihan Li

**Affiliations:** ^1^ Institute of Medical Biology Chinese Academy of Medical Sciences and Peking Union Medical College Yunnan Key Laboratory of Vaccine Research and Development on Severe Infectious Diseases Kunming 650118 China; ^2^ Stemirna Therapeutics Co., Ltd. Shanghai China; ^3^ National Institute for Viral Disease Control and Prevention China CDC Beijing China

1

Dear Editor,

More than 5.75 million deaths and 396.55 million cases of coronavirus disease 2019 (COVID‐19) have been reported, including those caused by at least five variants of severe acute respiratory syndrome coronavirus 2 (SARS‐CoV‐2).^1^ This situation has caused public concern about the effectiveness of immune responses to emerging variants elicited by boosters of different vaccines in previously immunized populations.^2^, ^3^ Our study, based on a cohort immunized with two doses of the inactivated vaccine and boosted with the messenger RNA (mRNA) vaccine at an interval of 6 months, investigates immunity of heterologous vaccination. The inactivated and mRNA vaccines used in this study were developed based upon the sequence of the Wuhan strain.^4^, ^5^ In total, 288 healthy adult volunteers were immunized with an inactivated vaccine (150 U/dose) at an interval of 14 days; neutralizing antibodies were identified as having a geometric mean titer (GMT) of 2.1 and as negative in 52% of this population at 6 months after full immunization. Among them, 118 and 97 immunized subjects were boosted with the inactivated (150 U/dose) or mRNA (25 μg/dose) vaccine, and 73 immunized subjects were monitored as non‐boosted controls. The average ages of the three groups were 43.04 ± 13.05, 31.86 ± 7.62 and 30.05 ± 7.03 years, respectively (Table S1). Immunological observation was until day 180 post‐boost, which was available for 60, 61 and 68 subjects in the inactivated vaccine boost, mRNA vaccine boost and nonboosted groups, respectively (Figure S1). Antibody assays performed at days 14 and 180 post‐boost showed GMTs of 829 and 455 on days 14 and 180, respectively, against the Wuhan strain in the mRNA boost group, in comparison to GMTs of 39 and 19, respectively, in the inactivated vaccine boost group (Figure 1). Binding antibody titers reached 71864 and 29675 and 3162 and 1993 on days 14 and 180, respectively, in the mRNA and inactivated vaccine boost groups (Figure 1). Further cross‐neutralization for the B.1.617.2 and B.1.1.529 strains suggested that sera from the mRNA vaccine boost group possess a strong neutralizing antibody titer against the B.1.617.2 strain, with GMTs reaching 540 and 335 at days 14 and 180 post‐boost, respectively. The data are close to those against the Wuhan strain; sera showed GMTs of 108 and 33 against the B.1.1.529 strain at the same times (Figure 2). Analysis of dynamic variation of these neutralizing antibodies against variants in 17 subjects from the three groups suggested that non‐boosted individuals displayed a continuous decline in neutralizing antibodies during the study to titers of 1 (Figure 3) and that the inactivated vaccine‐ and mRNA vaccine‐boosted groups presented a tendency to decline from high levels (Figure 3). However, there was distinct differential variation in the neutralizing antibody titer in the inactivated vaccine‐boosted and mRNA vaccine‐boosted groups. The antibody titer in individuals boosted by the mRNA vaccine showed only a slight decline and even remained stable in one individual (Figure 3), whereas the titer variation among those boosted by the inactivated vaccine presented the same slope as those of the non‐boosted group. Importantly, these tendencies were similar among antibodies against the Wuhan, B.1.617.2 and B.1.1.529 strains (Figure 3). These data highlight a possible vaccine strategy that might achieve an increased antibody response against current variants in inactivated vaccine‐immunized individuals with mRNA vaccine booster immunization that might be capable of maintaining effective immunity for at least 6 months, enhancing confidence in the defence against the COVID‐19 pandemic, even though the immunological mechanism underlying this phenomenon is currently unclear.

**FIGURE 1:**
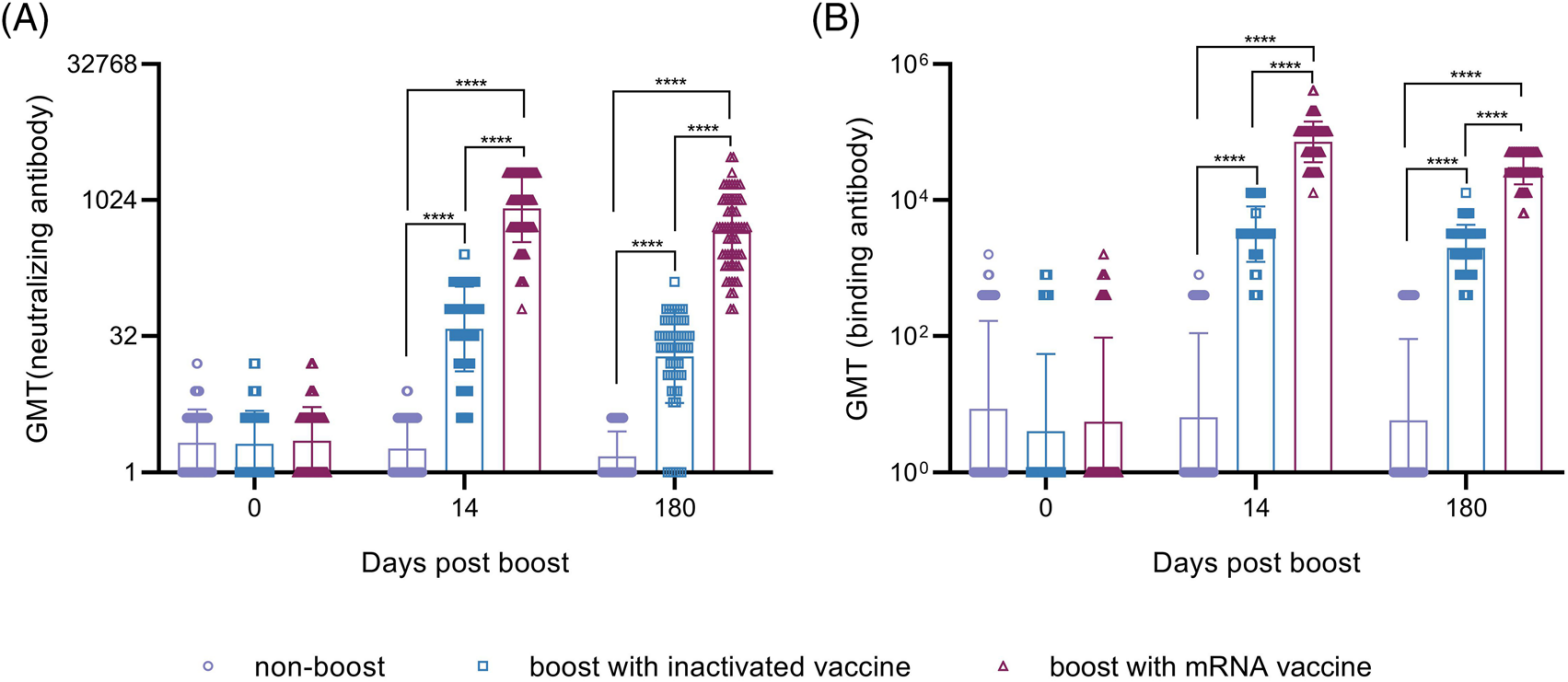
Production of antibodies against the Wuhan strain and binding antibodies was induced in all individuals immunized. (A) Neutralizing antibodies against the Wuhan strain at 14 and 180 days after booster immunization. (B) Binding antibodies (IgG) against the S1 protein at 14 and 180 days after booster immunization. A non‐boosted group, an inactivated vaccine‐boosted group and an mRNA vaccine‐boosted group were included. GMT denotes the geometric mean titers of the antibody. *.01 < *p* < .05; **.001 < *p* < .01; ***p < .001; ****p < .0001; GMT, geometric mean titer; ns, not significant. *Significant when compared between groups

**FIGURE 2:**
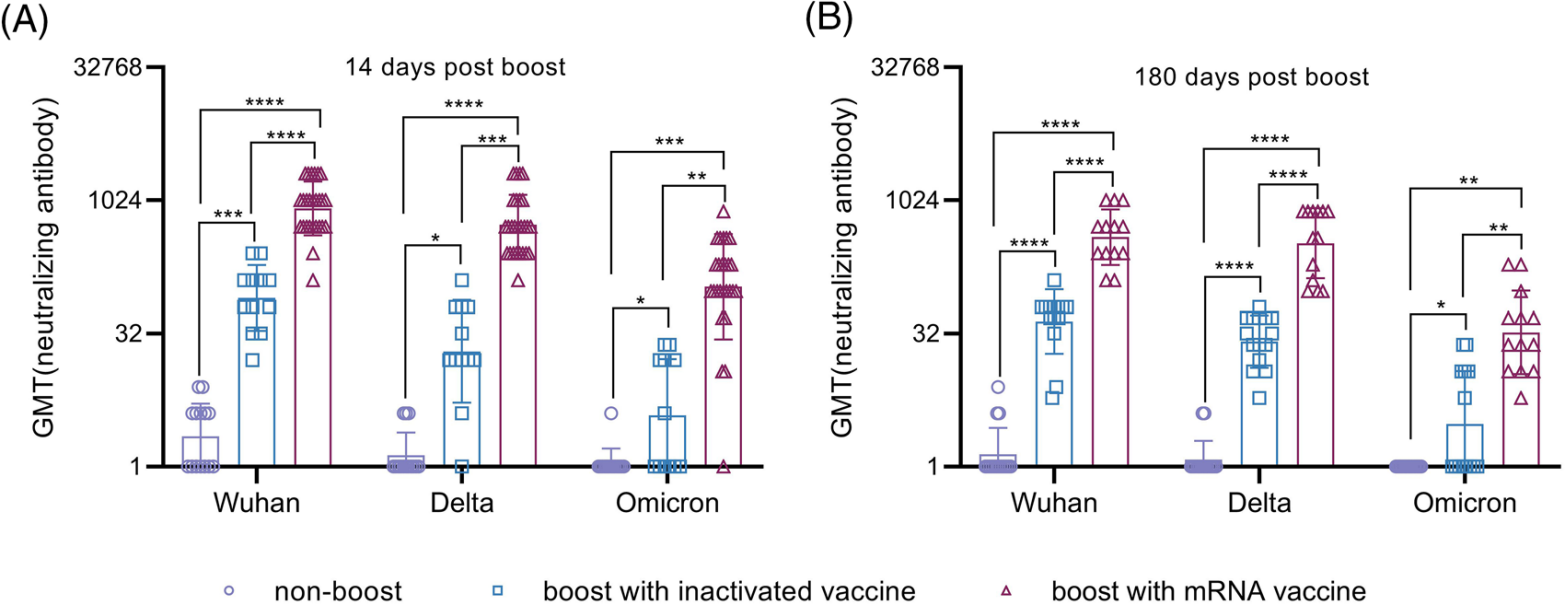
Cross‐neutralizing antibodies against the B.1.617.2 and B.1.1.529 strains in randomly selected immunized individuals. (A) Neutralizing antibodies against the B.1.617.2 and B.1.1.529 strains at 14 days after booster immunization. (B) Neutralizing antibodies against the B.1.617.2 and B.1.1.529 strains at 180 days after booster immunization. GMT denotes the geometric mean titers of the antibody.*.01 < *p* < .05; **.001 < *p* < .01; ***p < .001; ****p < .0001; GMT, geometric mean titer; ns, not significant. *Significant if compared between groups

**FIGURE 3:**
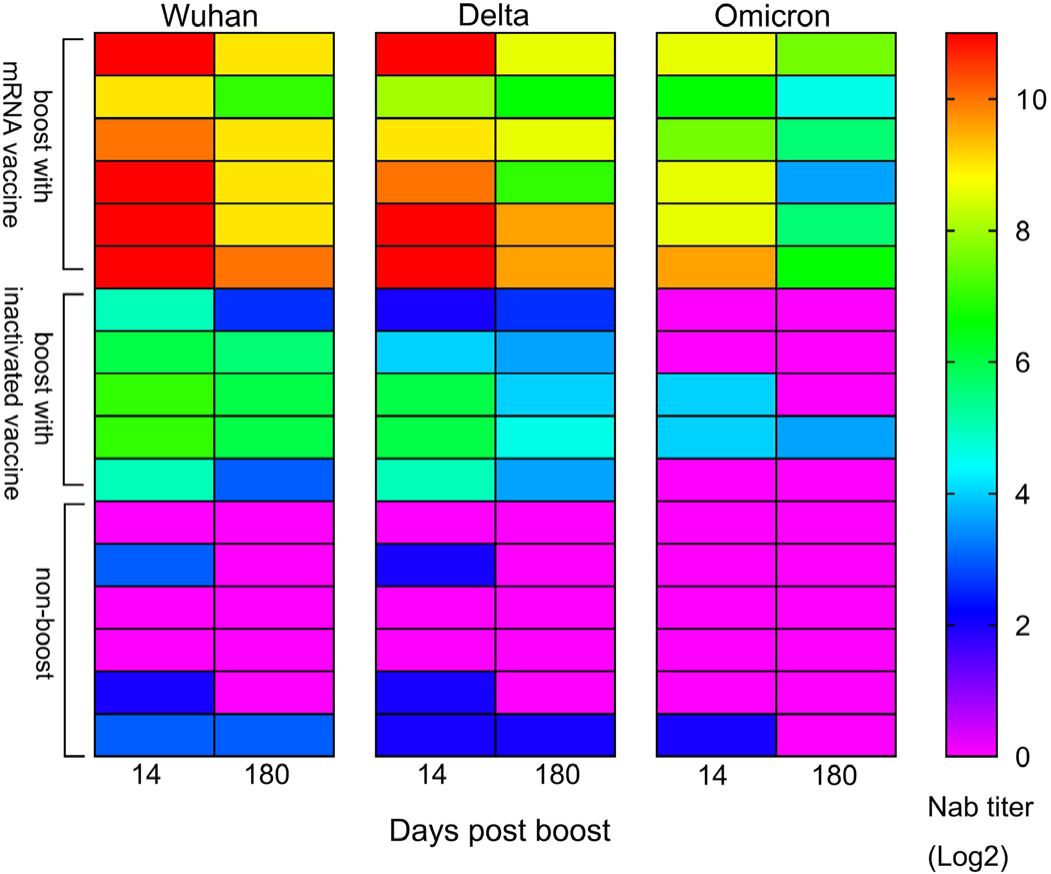
Dynamic alteration of neutralizing antibodies against the Wuhan, B.1.617.2 and B.1.1.529 strains in the same individuals. A non‐boosted group (*n* = 6), an inactivated vaccine‐boosted group (*n* = 5) and an mRNA vaccine‐boosted group (*n* = 6) were included

The S protein‐specific T cell response assay using viral receptor‐binding domain (RBD) peptides with specific mutations identified in the B.1.1.7, B.1.617, B.1.617.2, B.1.351, B.1.1.529.BA.1 and Wuhan strains suggest higher spot numbers for the wild‐type RBD of the S protein than for the RBDs derived from the variants at day 28 post‐boost in individuals boosted with the mRNA vaccine (Figure 4A). However, spot counting in the assay at day 180 post‐boost indicated similar effective T cell responses against the RBD peptide with the mutations of B.1.1.529.BA.1 in comparison to those against RBD peptides with characterized mutations of previous variants (Figure 4B). These results suggest that activated immune memory is definite in individuals primarily immunized with an inactivated vaccine and boosted with an mRNA vaccine. According to clinical monitoring for 28 days after boosting, mild (grade 1) to moderate (grade 2) pain, redness, swelling and pruritus at the injection site were reported as the main local adverse events over 7 days. Approximately 26 subjects reported severe pain (Table S2). Transient fatigue was the most common systemic event, with 21% of the participants in this group reporting this adverse event. Nine subjects reported transient fever (grade 2) with general weakness or headache. The incidence rate of adverse reactions in the mRNA group was obviously lower than the rates reported in clinical studies of other mRNA vaccines.^6^

**FIGURE 4:**
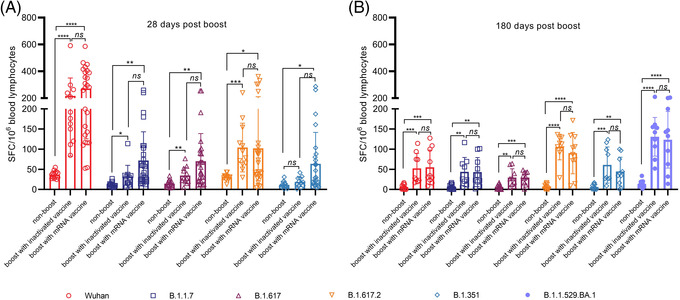
Antigen‐specific effective T cell responses (IFN‐γ ELISpot) against several RBD proteins in randomly selected immunized individuals. IFN‐γ‐positive effective T cell responses against the wild‐type RBD peptide of Wuhan strain, an RBD peptide containing the N501Y mutation of B.1.1.7, an RBD peptide containing the L452R and E484Q mutations of B.1.617, an RBD peptide containing the L452R and T478K mutations of B.1.617.2, an RBD peptide containing the K417N, E484K and N501Y mutations of B.1.351, an RBD peptide containing the G339D, S371L, S373P, S375F, K417N, N440K, G446S, S477N,T478K, E484A, Q493R, G496S, Q498R, N501Y and Y505H mutations of B.1.1.529.BA.1 strain in different groups. (A) IFN‐γ‐positive effective T cell responses against different mutant proteins at 28 days after booster immunization. (B) IFN‐γ‐positive effective T cell responses against different mutant proteins at 180 days after booster immunization. *.01 < *p* < .05; **.001 < *p* < .01; ***p < .001; ****p < .0001; IFN, interferon; ns, not significant; RBD, receptor‐binding domain

Clinical trials of inactivated vaccines report antigen‐specific humoural and cellular immune responses and protective efficacy against pandemics caused by the Wuhan strain.^7^, ^8^ However, subsequent studies on the duration of immunity elicited by the vaccine against the Wuhan strain and other variants indicate dramatic declines in neutralizing and binding antibody titers, suggesting that a booster vaccination is needed to maintain neutralizing antibodies in this vaccinated population.^9^ In this study, a dose of 25 μg of mRNA vaccine as a clinical‐stage product was used for booster immunization in individuals previously immunized with an inactivated vaccine, eliciting robust enhancement of antigen‐specific antibody titers and cellular immune responses, which is similar to the data that heterologous boosting is associated with more robust immune responses than homologous boosting.^10^ Indeed, they were substantially greater than those achieved with booster immunization with the inactivated vaccine in our previous work and, importantly, also exhibited cross‐reactivity with an antigen carrying the mutations of the delta and omicron variants, showing not only positive specific effective T cell responses against S antigens with various mutations but also high neutralizing antibody titers against the B.1.617.2 and B.1.1.529 and Wuhan strains.

In conclusion, all of these data support the conclusion that boosting previously inactivated vaccine‐immunized individuals with an mRNA vaccine is a promising strategy for increasing population‐level immunity against the rising pandemic caused by variants.

## CONFLICT OF INTEREST

The authors declare no competing interests.

## Supporting information

Supporting Information
