# Supplementary material for: Immunological evaluation of an mRNA vaccine booster in individuals fully immunized with an inactivated SARS‐CoV‐2 vaccine
Source: Clin Transl Med. 2022 Jun 9;12(6):e875. doi: 10.1002/ctm2.875 (PMC9178391; doi:10.1002/ctm2.875)
Supplement: Supplementary file 1 — Supporting Information [file CTM2-12-0-s001.docx]

Table S1.Demographic characteristics of enrolled participants.

| Groups | Non-boost (n=73) | Boost with inactivated vaccine (n=118) | Boost with mRNA vaccine (n=97) |
| --- | --- | --- | --- |
| Age, years | | | |
| 18-30 | 19(26.0%) | 51(43.2%) | 64(66.0%) |
| 31-40 | 15(20.5%) | 54(45.8%) | 24(24.7%) |
| 41-50 | 6(8.2%) | 11(9.3%) | 7(7.2%) |
| 51-60 | 33(45.2%) | 2(1.7%) | 2(2.1%) |
| Mean | 43.04(13.05) | 31.86(7.62) | 30.05(7.03) |
| Sex | | | |
| Male | 34(46.6%) | 77(65.3%) | 58(59.8%) |
| Female | 39(53.4%) | 41(34.7%) | 39(40.2%) |
| Data are n(%) or mean(SD). There was no significant difference in age among the three groups. | | | |

Table S2: Local and Systemic Reactions Reported within 7 Days.

| All adverse reactions | | | |
| --- | --- | --- | --- |
|  | Boost with mRNA vaccine (n=92) | Boost with inactivated vaccine cohort(n=116) | p value |
| Local adverse reactions | | | |
| Pain | 53(57%) | UN | <0.0001 |
| Grade1 | 27(29%) | UN | … |
| Grade2 | 26(28%) | UN | … |
| Systemic adverse reactions | | | |
| Fever | 26(28%) | UN | <0.0001 |
| Grade1 | 17(18%) | UN | … |
| Grade2 | 9(10%) | UN | … |
| Fatigue | 20(22%) | UN | <0.0001 |
| Grade1 | 9(10%) | UN | … |
| Grade2 | 11(12%) | UN | … |
| Data are n (%). Any refers to all the participants with any grade adverse reactions or events. UN：non-reported. | | | |

**Figure legends**

Figure S1: Enrollment and inclusion in the study.

The diagram represents all enrolled participants in June 2021. A total of 288 subjects were enrolled in the study. Two participants in the nonboosted group, five in the mRNA vaccine-boosted group and two in the inactivated vaccine-boosted group declined further participation after injection. In total, 279 subjects were involved in blood collection at 14 days post immunization and in the monitoring of adverse reactions and events. A total of 189 subjects were involved in blood collection at 180 days post immunization.
